# The Expanding Armamentarium of Innovative Bioengineered Strategies to Augment Cardiovascular Repair and Regeneration

**DOI:** 10.3389/fbioe.2021.674172

**Published:** 2021-06-01

**Authors:** Stefan Elde, Hanjay Wang, Y. Joseph Woo

**Affiliations:** ^1^Department of Cardiothoracic Surgery, Stanford University, Stanford, CA, United States; ^2^Stanford Cardiovascular Institute, Stanford University, Stanford, CA, United States; ^3^Department of Bioengineering, Stanford University, Stanford, CA, United States

**Keywords:** regeneration, heart regeneration, cardiac regeneration, myocardial regeneration, bioengineering, cell sheet and tissue engineering, hydrogel, inflammation

## Abstract

Cardiovascular disease remains the leading cause of death worldwide. While clinical trials of cell therapy have demonstrated largely neutral results, recent investigations into the mechanisms of natural myocardial regeneration have demonstrated promising new intersections between molecular, cellular, tissue, biomaterial, and biomechanical engineering solutions. New insight into the crucial role of inflammation in natural regenerative processes may explain why previous efforts have yielded only modest degrees of regeneration. Furthermore, the new understanding of the interdependent relationship of inflammation and myocardial regeneration have catalyzed the emergence of promising new areas of investigation at the intersection of many fields.

## Introduction

Cardiovascular disease is responsible for 17.6 million deaths worldwide every year, and the cost of treating these patients is expected to double over the next two decades (Heidenreich et al., 2011; Benjamin et al., 2019). Significant advancements in revascularization strategies after myocardial infarction (MI) such as coronary artery bypass grafting (CABG) and percutaneous coronary intervention (PCI) have considerably improved outcomes, but do not address microvascular perfusion deficits that result in adverse ventricular remodeling despite successful macrorevascularization (Araszkiewicz et al., 2006). This unmet clinical need has stimulated a significant interest in bioengineering strategies including molecular and cellular, tissue, biomaterial, and biomechanical engineering. In this mini review, we will briefly discuss current strategies, challenges, and future directions.

Given the exponential expansion of new techniques that fall under the broad definition of bioengineering, for the purpose of this article we find it useful to define the scope of *bioengineered strategies* that will be covered in this review.

### Molecular and Cellular Bioengineering

Application of engineering principles at the molecular and cellular levels such as the development of novel cytokines, targeted delivery of intracellular cargo, modulation of gene expression, and cross-species photosynthetic oxygen production.

### Tissue Engineering

Engineered solutions to recapitulate viable myocardium from myocardial patches, cell sheets, and engineered extracellular matrices embedded with various cell types.

### Biomaterial Engineering

Engineering strategies involving hydrogels, cellular scaffolds, or other insoluble substrates that are either impregnated with progenitor cells, growth factors, cytokines, or possess other proangiogenic stimulatory cues.

### Biomechanical Engineering

Engineered substrates that mimic the anisotropic properties of native myocardium and thereby promote the proper alignment of myocardial fibers.

## Molecular and Cellular Bioengineering

Molecular bioengineering techniques to develop novel analogs of endogenous cytokines are a powerful tool to modulate the activation and suppression of specific pathways relevant to the regenerative response (Table 1). Following significant insult such as MI, an influx of inflammatory cytokines triggers an acute inflammatory response and migration of macrophages, fibroblasts, and T cells to the infarct zone. Remodeling of the extracellular matrix (ECM) and secretion of potent chemo attractants such as stromal cell-derived factor 1 (SDF-1α) recruit endothelial progenitor cells (EPCs) to the border zone to initiate angiogenesis and myocardial regeneration in rodents (Ingason et al., 2018). The pro-angiogenic properties of SDF-1α and its conservation across many species made it an appealing target for inducing natural myocardial angiogenesis and regeneration. For these reasons, Hiesinger et al. (2011) used molecular modeling to create a synthetic Engineered SDF-1α Analog (ESA) that demonstrated enhanced stability and efficiency in microrevascularization in a murine ischemic cardiomyopathy model. ESA was subsequently shown to improve angiogenesis and perfusion in a rat hindlimb ischemia model (Edwards et al., 2016) and an ovine MI model (MacArthur et al., 2013a).

**TABLE 1:** *In vivo* molecular and cellular engineering approaches to myocardial regeneration.

Author, year of publication	Model	Therapy	Delivery route	Dose	Outcomes
Ingason et al., 2018	P1 mouse, apical resection	N/A	N/A	N/A	Regeneration in neonatal mice, proof of concept
Hiesinger et al., 2011	Mouse, LAD ligation	ESA	Intramyocardial injection	6 μg/kg ESA	Increased EF, CO, SV, fractional area change
Edwards et al., 2016	Rat, hind limb ischemia	ESA	Quadricep injection	6 μg/kg ESA	Increased perfusion ratio by doppler/Increased capillary density/Increased VEGF MRNA
MacArthur et al., 2013a	Sheep, LAD ligation	ESA	Intramyocardial injection	6 μg/kg ESA	Improved ventricular function Increased EPC chemotaxis Increased capillary and arteriolar density Decreased infarct size Increased maximal principle strain Steeper slope of end systolic pressure volume relationship
Mulyasasmita et al., 2014	Mouse, hindlimb ischemia	hiPSC-ECs VEGF	Protein-polyethylene glycol hydrogel	5 × 10^5^ cells 3 μg VEGF	Reduced inflammation Increased muscle regeneration
Cai et al., 2015	Mouse, subcutaneous injection	hASC	Protein polyethylene glycol hydrogel	5 × 10^5^ cells	Improved cell survival and retention
Steele et al., 2020	Mouse, LAD ligation Sheep, LAD ligation	ESA HGFdf	Hyaluronic acid hydrogel with PEG-PLA nanoparticles	ESA 25 μg 16 μg HGFdf	Reduction in scar size Increased density of borderzone arterioles Improved ventricular function and geometry
Schoger et al., 2020	Mouse	CRISPR-mediated gene activation	Adeno-associated virus serotype 9	N/A	Proof of concept, enhanced gene expression of *mef2d* and *Klf15*
Wang et al., 2020b	P1 Rat, LAD ligation	N/A	N/A	N/A	Regeneration in neonatal rats, proof of concept
Vagnozzi et al., 2020	Mouse, LAD ligation	MNCs CPCs Zymosan	Intramyocardial injection	150,000 MNCs or CPCs 10–20 μg zymosan	Inflammation stimulates improved ventricular performance
Santoso et al., 2020	Mouse, LAD ligation	Induced cardiomyocyte exosomes	Intramyocardial injection	4 × 10^8^ exosomes	Preserved ventricular performance Increased cardiomyocyte viability
Cohen et al., 2017	Rat, LAD ligation	Cyanobacteria	Intramyocardial injection	1 × 10^6^ *Synechococcus elongatus* cells	Improved tissue oxygenation 60% increase in cardiac output vs. control Improved EF 4-weeks post MI
Schenck et al., 2015	Mouse, full thickness skin defect	Microalgae (*Chlamydomonas reinhardtii*)	*Integra matrix double layer scaffold	1 × 10^4^ *C. reinhardtii* cells	Chimeric tissues of *C. reinhardtii* and mouse cells Viable algae at 5 days
Chávez et al., 2016	Mouse, full thickness skin defect	Genetically modified (+VEGF) microalgae (*Chlamydomonas reinhardtii*) HUVECS	*Integra dermal regeneration template	Variable	No significant adverse immune response Successful expression of VEGF via *C. reinhardtii*

Summary of *in vivo* studies investigating molecular and cellular engineering solutions for myocardial regeneration or treatment of ischemic heart disease. Note that the outcomes column is an abbreviated summary of the findings relevant to the focus of this review and is not intended to summarize the study as a whole. Left anterior descending coronary artery (LAD), engineered stromal cell-derived factor-1a (ESA), ejection fraction% (EF), cardiac output (CO), stroke volume (SV), vascular endothelial growth factor (VEGF), end diastolic volume (EDV), end systolic volume (ESV), endothelial progenitor cell (EPC), human induced pluripotent stem cells (hiPSC), human adipose-derived stem cell (hASC), poly(ethylene glycol)-block-poly(lactic acid) (PEG-PLA), dimeric fragment of hepatocyte growth factor (HGFdf), bone marrow mononuclear cells (MNCs), cardiac mesenchymal cells/cardiac progenitor cells (CPCs), human umbilical vein endothelial cells (HUVECS), *Integra Life Science Corporation, Plainsboro, NJ, United States.

**FIGURE 1:**
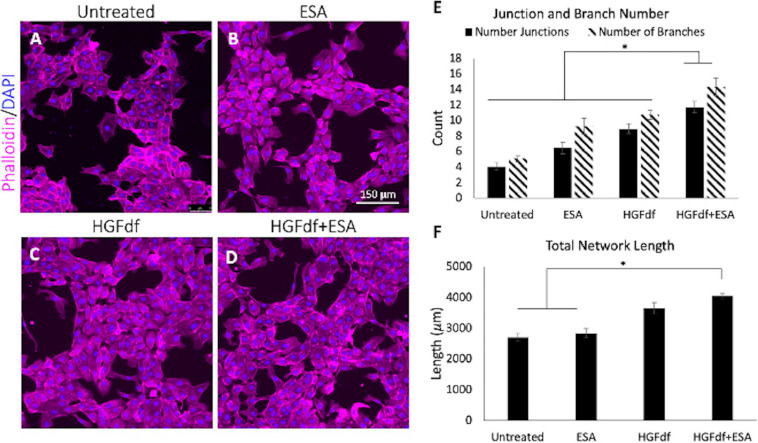
Assessment of *in vitro* angiogenesis. Human umbilical cord vein endothelial cells were treated with **(A)** untreated, **(B)** engineered stromal cell-derived factor 1α(ESA), **(C)** engineered dimeric fragment of hepatocyte growth factor (HGFdf) or **(D)** a combination of HGFdf and ESA. The extent of network formation **(E,F)** was evaluated in all groups. Pairwise student *t*-test with Bonferroni’s correction, *p < 0.05 (Steele et al., 2020).

Direct intramyocardial injection of cytokines or growth factors has proven to be inefficient due to their susceptibility to rapid degradation and diffusion away from the target site. To address these challenges, one group developed a shear thinning hydrogel to serve as the vehicle for cytokine or stem cell delivery via a catheter and returns to its gel form post-injection, named Shear-Thinning Hydrogels for Injectable Encapsulation and Long-Term Delivery (SHIELD) (Mulyasasmita et al., 2014; Cai et al., 2015). Using this novel hydrogel to encapsulate another bioengineered analog of a potent proangiogenic and antiapoptotic cytokine, dimeric fragment of hepatocyte growth factor (HGFdf), resulted in sustained HGFdf release and improved ventricular function with evidence of enhanced angiogenesis in a mouse model (Steele et al., 2020). Combining multiple engineered cytokines, specifically ESA + HGFdf, has also proven effective at reducing scar size and improving angiogenesis after MI in both a small animal model and in sheep (Figure 1; Steele et al., 2020).

The success of these efforts to engineer biologically active, shelf-stable, pro-angiogenic small molecules suggests that *in vivo* modulation of the pathways that govern natural regenerative pathways may be possible in the near future. For example, a recent study from Schoger et al. (2020) demonstrated the feasibility of using CRISP/Cas9 gene editing *in vivo* to modify cardiomyocyte (CM) gene expression in a mouse model. Neonatal mice, piglets, and rats all exhibit the capacity for natural myocardial regeneration after myocardial infarction, which is an encouraging sign that these strategies may be translatable to humans pending further study (Wang et al., 2020b).

Complex processes such as the transient ability of neonatal mammals to regenerate injured myocardium are rarely regulated by a single gene or pathway. Accordingly, myocardial regeneration in mammals is a highly regulated process that depends on a symphony of mediators (Desgres and Menasché, 2019). For this reason, one limitation of molecular engineering techniques is that activating a single pathway *in absentia* a coordinated cellular response may result in incomplete or partial activation of the regenerative response. A related challenge of modulating CM developmental pathways via bioengineered small molecules is balancing the specificity of the effectors to mitigate undesirable off-target effects, while attempting to also activate the necessary ancillary or supportive pathways required for regeneration.

For decades, stem cells appeared to be the intuitive solution to the puzzle of myocardial regeneration. However, there is strong evidence to suggest that the mild therapeutic benefit of cell therapy for treatment of ischemic heart disease is actually due to an acute sterile inflammatory response (Vagnozzi et al., 2020). In this study by Vagnozzi et al. (2020), killed cardiac progenitor cells induced an inflammatory response that attenuated fibrosis and rescued ventricular function. Although an acellular inflammatory agent, Zymosan, had a similar effect, it appeared as though cellular debris such as the micro-RNA (miRNA) contained within exosomes may provide a potentially intervenable entry point into the regulatory mechanisms of regeneration. To address this, multiple groups have turned to exosomes from induced pluripotent stem cells (iPSC) that have transdifferentiated into CMs. Exosomes are an appealing vehicle for delivery of a balanced milieu of endogenous miRNA, peptides, and other small molecules to provide the environmental cues to the resident cells of the myocardium. A recent study found that injection of exosomes derived from induced CMs both reduced apoptosis and fibrosis while also upregulating autophagy of cellular debris in the infarcted territory, a necessary prelude to full scale microrevascularization (Santoso et al., 2020). Similar effects have been demonstrated with extra cellular vesicles (EVs), which contain exosomes and a variety of small signaling molecules (Menasché, 2018).

Recent discoveries resulting from innovative approaches in cellular engineering have the potential to create entirely new fields of research. One example of innovative cellular engineering is from Cohen et al. (2017), who demonstrated that administering cyanobacteria into the ischemic rodent heart significantly improves oxygen delivery and ventricular performance after MI. This concept has been reproduced by other groups that have shown the ability of other photosynthetic bacteria to attenuate the murine fibroblast response to hypoxia, and to switch ischemic rat CMs from anaerobic to aerobic metabolism (Hopfner et al., 2014; Haraguchi et al., 2017). Studies to optimize the stability of photosynthetic bacteria *in vivo* by introducing them via scaffolds or fibrin based hydrogels have successfully reduced cell scattering and proven effective in wound healing assays (Schenck et al., 2015; Chávez et al., 2016; Wang et al., 2019b). Furthermore, the genetic adaptability of cyanobacteria allows for essentially limitless creativity in modifying or augmenting gene expression, such as enhancing expression of angiogenic growth factors like vascular endothelial growth factor (VEGF) (Chávez et al., 2016). These findings have widespread implications for fields such as tissue engineering, organ preservation and transplantation, wound healing, diabetic complications, and neurovascular disease (Wang et al., 2019b).

## Tissue Engineered Solutions

Engineered cardiac muscle patches are an emerging potential therapy to address the microvascular perfusion deficit following ischemic insult, e.g., after (MI) (Table 2). Patches (also referred to in the literature as scaffolds) may be comprised of reconstituted synthetic materials such as polymers or metals, or as naturally occurring materials such as collagen, chitosan, or alginate, among many others (Cui et al., 2016). Typically, these constructs are applied directly to the epicardium, providing mechanical support to attenuate adverse myocardial remodeling such as wall thinning and fibrosis (Serpooshan et al., 2013). In addition to mechanical reinforcement of the myocardium, patches may also be engineered to serve as a cellular substrate (i.e., engineered ECM) to recruit and retain cell types involved in native myocardial regeneration and angiogenesis (Serpooshan et al., 2013). Building on these techniques, scaffolds can serve as vehicles to deliver therapeutic cytokines, growth factors, proteins, and stem cells to the affected areas (Naveed et al., 2018).

**TABLE 2:** *In vivo* tissue engineering approaches to myocardial regeneration.

Author, year of publication	Model	Therapy	Delivery route	Scaffold	Dose	Outcomes
Serpooshan et al., 2013	Mouse, LAD ligation	Mechanical support of ischemic myocardium	Grafted onto ischemic epicardium	Acellular 3D collagen (type I) patch with elastic moduli 2–10 kPa	N/A	Improved EF and FS Enhanced neo-angiogenesis Diminished fibrosis Migration of native cardiac cells into patch
Chachques et al., 2008	Human, ischemic cardiomyopathy	Autologous BMCs	Intramyocardial injection during CABG ± BMC seed collagen matrix	*CE Mark collagen kit	250 ± 28 million cells	Safe and feasible No difference in arrhythmias Attenuated adverse ventricular remodeling
Menasché et al., 2018	Human, ischemic cardiomyopathy	hESC derived cardiac progenitor cells	Epicardial patch during CABG	Fibrin patch	5–10 million cells	Safe and feasible No difference in frequency of tumors or arrhythmias 50% alloimmunization
Huang et al., 2020	Rat, LAD ligation Pig, LAD ligation	Synthetic cardiac stromal cells	Epicardial patch	Decellularized porcine ECM + synthetic cardiac stromal cells	2 × 10^6^ cells	Improved EF and FS at 7 days (pig) and 3 weeks (rat) Reduced infarct size Increased capillary density Increased cardiomyocyte cell cycle activity
Shudo et al., 2013	Rat, LAD ligation	Aortic SMC and EPCs	Epicardial cell sheet	Bi-level cell sheet	1.3 × 10^6^ SMCs 1.3 × 10^6^ EPCs	Enhanced capillary density and functional microvasculature Migration of EPCs and SMCs into native myocardium Reduced adverse ventricular remodeling Improved EF and FS at 4 weeks post injury
Shudo et al., 2017	Rat, LAD ligation	Bone marrow derived SMC and EPCs	Epicardial cell sheet	Bi-level cell sheet	1.5 × 10^5^/cm^2^ EPCs 1.5 × 10^5^/cm^2^ SMCs	Improved EF Enhanced neovascularization Reduced adverse ventricular remodeling
von Bornstädt et al., 2018	Rat, femoral artery interposition graft	Human aortic SMCs and skin fibroblasts	Interposition graft	Bi-level cell sheet conduit	1.5 × 10^5^/cm^2^ SMCs	Rapid conduit maturation (2 weeks) Responsive to vasoactive agents 100% patency at 8 weeks Similar histological structure to native arteries

*Summary of *in vivo* studies investigating tissue engineering solutions for myocardial regeneration or treatment of ischemic heart disease. Note that the outcomes column is an abbreviated summary of the findings relevant to the focus of this review and is not intended to summarize the study as a whole. Left anterior descending coronary artery (LAD), ejection fraction% (EF), fractional shortening% (FS), bone marrow cells (BMC), coronary artery bypass grafting (CABG), ^∗^Pangen 2; Urgo Laboratory, Chenove, France, human embryonic stem cells (hESC), endothelial progenitor cells (EPC), smooth muscle cells (SMC).*

While cardiac patches embedded with pro-angiogenic cell types such as mesenchymal stem cells (MSCs), induced pluripotent stem cells (iPSCs), CMs derived from iPSCs, skeletal myoblasts, and cells derived from bone marrow continue to be investigated (Bw and Me, 2019), select trials in humans have had shown variable results (Chachques et al., 2008; Steele et al., 2017; Menasché et al., 2018). Specifically, engineering living patches introduces issues such as potential immunogenicity or tumorigenicity, transportation and storage logistics, and quality control concerns. Given these valid concerns, there has been a recent resurgence of interest in acellular approaches.

Combining many of the aforementioned techniques, one group recently developed a shelf-stable cardiac patch using decellularized porcine ECM embedded with polylactic-co-glycolic acid (PLGA) microparticles containing growth factors from cardiac stromal cells in a porcine model. By recapitulating native paracrine signaling while avoiding the inherent challenges of stem cell engraftment, this novel artificial cardiac patch preserved ejection fraction (EF), reduced pathologic remodeling, increased residual viable myocardial tissue, promoted angiogenesis, and may be stored for up to 28 days (Huang et al., 2020).

In contrast to engineering synthetic substrates to provide mechanical support to the infarcted myocardium while simultaneously stimulating angiogenesis, several groups have focused on repurposing nature’s preexisting efficiencies. Shudo et al. (2013) engineered a scaffold-free bilevel cell sheet comprised of EPCs and smooth muscle cells (SMCs) from the thoracic aorta which was applied to ischemic myocardium in a rat model. Fate-Tracking assays showed evidence of migration of the EPC/SMCs into the myocardium followed by a transition into mature and functional microvasculature (Shudo et al., 2013). Similarly, the same group found that ECM rich in fibronectin may help guide MSCs toward a SMC fate, suggesting that an MSC/ECM cell sheet may provide therapeutic benefit. Combining these two findings, they were able to develop a sheet derived entirely from bone marrow which enhances neovascularization, limits adverse remodeling, and improves ventricular function (Shudo et al., 2017). Collectively, these findings also have potential for clinical translation as vascular conduits, demonstrated by using tubularized cell sheets in a rat femoral artery interposition graft model (von Bornstädt et al., 2018). Importantly, the mechanical properties and specifically the stiffness of cell sheets can be easily modified by titrating the collagen content during incubation (Zhu et al., 2020).

## Biomaterial Engineered Solutions

In an effort to address the challenges of low cell retention and engraftment in techniques that utilize stem cells to repair injured myocardium (Laflamme et al., 2007; Terrovitis et al., 2010), injectable hydrogels have gained traction as a possible solution given their mechanical properties and 3D structure that may protect the fragile stem cells from membranous injury, host rejection, and cell death (Aguado et al., 2012; Dhingra et al., 2013) (Table 3). While injectable, shear-thinning hydrogels provide relative protection, optimizing the physical characteristics of the gel, both *ex vivo* during production and *in vivo* after injection, depends on the crosslinking strategy. As discussed above, SHIELD hydrogels were engineered to provide weak *ex vivo* interactions making injection possible, followed by significantly stronger crosslinking once exposed to temperatures above 34°C to maintain hydrogel integrity *in vivo* (Cai et al., 2015). There is ongoing debate regarding the optimal hydrogel stiffness, and this may vary depending on whether the intent is to provide mechanical support to the ventricular wall with or without stem cell transplantation or other cell therapies. Some studies suggest that intermediate stiffness gels (200–400 Pa) could promote the angiogenic potential of engrafted MSCs (Cai et al., 2016), while supraphysiologic gel stiffness may be optimal if the intent is purely mechanical support of the infarcted myocardial territory.

**TABLE 3:** *In vivo* biomaterial engineering approaches to myocardial regeneration.

Author, year of publication	Model	Therapy	Delivery route	Scaffold (if applicable)	Dose	Outcomes
Laflamme et al., 2007	Rat, LAD ligation	Human embryonic stem cell derived cardiomyocytes + pro-survival factors	Intramyocardial injection	N/A	10 × 10^6^ human embryonic stem cells	Limited adverse ventricular remodeling Preserved EF Partial remuscularization of infarct zone
Dhingra et al., 2013	Rat, LAD ligation	Allogeneic MSCs + Prostaglandin E2	Intramyocardial injection/hydrogel	Biodegradable hydrogel impregnated with prostaglandin E2	3 × 10^6^ cells	Improved MSC survival/immunoprivilege Improved ventricular FS and attenuated adverse remodeling
Cai et al., 2016	Mouse, subcutaneous injection	hASCs + hydrogel	Intramyocardial injection/hydrogel	SHIELD hydrogel, 200–400 Pa	5 × 10^5^ cells	Enhanced cell retention
Cohen et al., 2014	Mouse, LAD ligation	NRG + hydrogel	Intramyocardial injection/hydrogel	Biodegradable hydrogel impregnated with NRG	2.5 μg NRG 3.33 × 10^5^/mL rat cardiomyocytes	Enhanced EF Increased myocardial thickness at infarct border zone
Cohen et al., 2020	Sheep, LAD ligation	NRG + hydrogel	Intramyocardial injection/hydrogel	Biodegradable hydrogel impregnated with NRG	100 μg NRG	Enhanced EF and contractility at 8 weeks Reduced infarct size
Purcell et al., 2012	Mouse, LAD ligation	rSDF-1α + hydrogel	Intramyocardial/hydrogel	Hyaluronic acid hydrogel	200 ng rSDF-1α	Enhanced BMC chemotaxis to remodeling myocardium

*Summary of *in vivo* studies investigating biomaterial engineering solutions for myocardial regeneration or treatment of ischemic heart disease. Note that the outcomes column is an abbreviated summary of the findings relevant to the focus of this review and is not intended to summarize the study as a whole. Left anterior descending coronary artery (LAD), ejection fraction% (EF), mesenchymal stem cells (MSC), fractional shortening% (FS), human adipose-derived stem cell (hASC), shear-thinning hydrogel for injectable encapsulation and long-term delivery (SHIELD), polyethylene glycol (PEG), neuregulin (NRG), recombinant stromal cell derived-factor-1a (rSDF-1α), bone marrow-derived cells (BMC).*

While mechanical support of the ischemic ventricular wall may facilitate later neovascularization, integration of biologically active substrates within the hydrogel may further augment angiogenesis and myocardial repair. One such example is Neuregulin (NRG), an epidermal growth factor with a critical role in CM development which has demonstrated utility in cardiomyopathy animal models. Analogous to the challenges of injectable therapies such as stem cells or other biologically active substances, recurrent infusions and off-target exposure preclude the clinical translation of an otherwise promising therapy. To address this, hydrogels encapsulating NRG were engineered to deliver a localized and sustained therapeutic dose while simultaneously providing mechanical support to the ischemic myocardium. This construct stimulated CM mitotic activity, reduced LV dilation, decreased infarct scar size, and enhanced ventricular function in mice and later in sheep 8 weeks post-MI (Cohen et al., 2014, 2020).

Utilizing the sustained, localized delivery of biologically active products via a hydrogel vehicle, similar approaches have shown promise with engrafted stem cells. A limitation of earlier technologies may have been that transplanted stem cells lose their immune privilege and are ultimately rejected upon prolonged interactions with the host myocardium (Dhingra et al., 2013). However, when hydrogels seeded with rat MSCs were treated with prostaglandin E2, which stimulates secretion of the cytokines CCL12 and CCL5, they retained their immune privilege and improved cardiac function in rats (Dhingra et al., 2013). These results stimulated interest in encapsulation of cytokines and exosomal cargo within the hydrogels, given the simplified production and scalability of this approach compared to using MSCs. Examples of cytokines and growth factors that have shown promise when integrated into hydrogels include stromal cell-derived factor-1 alpha (SDF-1α) (Purcell et al., 2012), insulin-like growth factor-1 (IGF-1), hepatocyte growth factor (HGFdf), and many others (Ferrini et al., 2019).

## Biomechanical Engineering

In healthy myocardium CMs use the ECM as an anchor for actomyosin to generate contractile force. In addition to the rapidly expanding library of small molecules that influence CM development and response after insult, mechanical cues also influence cell shape, protein expression, and differentiation (Engler et al., 2008) (Table 4). Engineered matrices that are too soft will provide inadequate resistance for the myosin power stroke, leading to inefficient myocardial contraction. Conversely, matrices that are too stiff lead to intracellular strain on protein structure and earlier loss of contractility when cultured with CMs. Unsurprisingly, it appears as though the optimal stiffness of engineered ECM is that which mimics *in vivo* ECM (Engler et al., 2008). This has implications for engineering solutions for myocardial regeneration and also provides insight into the mechanical dysfunction seen in pathologic states such as pathologic fibrosis following ischemic injury. This prompted investigation of the effect of proangiogenic peptides such as SDF-1α with respect to their mechanical effects on the injured myocardium. SDF-1α administration after MI appears to increase the elasticity of the border zone and strengthens the fibrotic myocardium, which may provide a mechanical advantage to CMs and attenuate adverse remodeling (Hiesinger et al., 2012). In addition to naturally occurring small molecules such as SDF-1α, engineered analogs such as ESA have demonstrated the ability to preserve biaxial mechanical properties of the native myocardium, improve myocardial relaxation, reduce infarct size, reduce ventricular thinning, and improve ventricular function (MacArthur et al., 2013b; Trubelja et al., 2014; Wang et al., 2019a).

**TABLE 4:** *In vivo* biomechanical engineering approaches to myocardial regeneration.

Author, year of publication	Model	Therapy	Delivery Route	Scaffold (if applicable)	Dose	Outcomes
Hiesinger et al., 2012	Mouse, LAD ligation	SDF-1α	Intramyocardial injection	N/A	6 μg/kg	SDF-1α treated peri-infarct myocardium with similar elasticity to normal ventricle SDF-1α treatment stiffened scarred ventricle
MacArthur et al., 2013b	Rat, LAD ligation	ESA	Intramyocardial injection	N/A	6 μg/kg	Enhanced EF and improved CO Reduced adverse remodeling Improved elasticity
Trubelja et al., 2014	Rat, LAD ligation	ESA	Intramyocardial injection	N/A	6 μg/kg	Increased relaxation rate and decreased transition strain
Wang et al., 2019a	Rat, LAD ligation	ESA	Intramyocardial injection	N/A	6 μg/kg	Greater wall thickness Reduced LVEDD Enhanced EF Reduced infarct size Preserved biaxial mechanical properties of left ventricle
Wang et al., 2020a	P1 mouse, LAD ligation	N/A	N/A	N/A	N/A	Natural myocardial regeneration in P1 mice results in similar biomechanical properties as the native myocardium
Notari et al., 2018	P3 mouse, apical resection	Local modification of ECM stiffness (BAPN, LOX inhibitor)	Oral administration	N/A	1 mg/mL	Decreasing stiffness of ECM results in extended window for natural regeneration in neonatal mice
Yu et al., 2018	Zebrafish, cryoinjury	N/A	N/A	N/A	N/A	Regenerating myocardium requires biomechanical stimulation

*Summary of *in vivo* studies investigating biomechanical engineering solutions for myocardial regeneration or treatment of ischemic heart disease. Note that the outcomes column is an abbreviated summary of the findings relevant to the focus of this review and is not intended to summarize the study as a whole. Stromal cell-derived factor-1α, engineered stromal cell–derived factor-1a analog (ESA), ejection fraction% (EF), cardiac output (CO), left ventricular end-diastolic dimension (LVEDD), 3-aminopropionitrile (BAPN, an inhibitor of the LOX ECM crosslinking enzyme), extracellular matrix (ECM).*

While biomechanical approaches to emulate the properties of native myocardium have shown promise and should continue to be investigated, naturally regenerated myocardium in a neonatal mouse MI model successfully replicates the mechanical properties of native uninjured myocardium (Wang et al., 2020a). Furthermore, studies in zebrafish have demonstrated that naturally regenerating myocardium is dependent on biomechanical stimulation, i.e., strain, to recover ventricular function after cryoinjury. Collectively, this evidence suggests that biomechanical cues such as ECM stiffness play an important role in the coordination of the regenerative response (Notari et al., 2018; Yu et al., 2018).

## Limitations of Current Techniques

The most challenging limitation to molecular and cellular engineering solutions are that profibrotic, inflammatory, and natural regenerative pathways have complex networks of built-in checks and balances which are difficult to precisely modulate. For example, in reference to regeneration, Berry et al. (2019) describe a “Goldilocks zone” of innate immune signaling, outside of which attempts at cellular reprogramming may be impaired. Additionally, because most molecular and cellular engineering solutions focus on endogenous pathways, the primary safety concerns relate to the potential for non-specific off-target effects. The principal safety concern of cell therapy and tissue engineering are rejection and the inherent potential for uncontrolled proliferation of pluripotent cells. Because exosomes are acellular, they are less immunogenic and have fewer safety concerns than transplantation of allogeneic progenitor cells (Gallet et al., 2017). Additionally, optimizing the delivery substrate without sacrificing cell retention remains a challenge. Direct application of a myocardial patch or hydrogel via a surgical operation are being replaced with catheter-injectable hydrogels, which should improve the safety profile from a periprocedural complication perspective (Steele et al., 2020). Although the chief concern with biomaterials is biocompatibility, most scaffolds and hydrogels in the current era are constructed from immunologically inert materials such as decellularized ECM, alginate, collagen, hyaluronan, fibrin, or insoluble polymers and appear to be safe (Seif-Naraghi et al., 2013; Cai et al., 2015).

## Conclusion

Despite significant advancements in our understanding and treatment of ischemic heart disease, the global burden and cost of treating these patients continues to increase. Bioengineering strategies to address the unmet need for paradigm-shifting therapies for ischemic heart disease have shown significant potential for clinical translation and are already being tested in large animal models. New insight into the potential therapeutic mechanism of cell therapy trials have lent credence to the theory of inflammation playing a central role in the natural regenerative pathways, which have informed future directions of this important research. It has become clear that successful translation of bioengineering solutions to treating ischemic heart disease will require an intricate and coordinated series of biologic and mechanical cues to replicate the robust myocardial regenerative pathways that occur naturally in neonatal mammals.

## Author Contributions

YJW and HW conceptualized the manuscript. SE wrote the manuscript. YJW and HW revised the manuscript. All the authors contributed to the article and approved the submitted version.

## Conflict of Interest

The authors declare that the research was conducted in the absence of any commercial or financial relationships that could be construed as a potential conflict of interest.
